# Gastrointestinal strictures in a pediatric patient with Satoyoshi syndrome

**DOI:** 10.1002/jpr3.70128

**Published:** 2025-12-12

**Authors:** Katherine (Tusia) Pohoreski, Gary Galante, Kiera Pajunen, Marie‐Anne Brundler, Samarjeet Bhandal, Iwona Wrobel

**Affiliations:** ^1^ Department of Pediatrics, Section of Pediatric Gastroenterology, Hepatology and Nutrition, Alberta Children's Hospital University of Calgary Calgary Alberta Canada; ^2^ University of Calgary Calgary Alberta Canada; ^3^ Departments of Pathology, Laboratory Medicine and Pediatrics and Alberta Precision Labs University of Calgary Calgary Alberta Canada; ^4^ Department of Radiology, Alberta Children's Hospital University of Calgary Calgary Alberta Canada

**Keywords:** alopecia, diarrhea, dystonia, fibrosis, growth failure

## Abstract

We present a novel case of gastrointestinal strictures in a young girl with Satoyoshi syndrome (SS), highlighting multi‐system features of alopecia universalis, painful muscle cramps with dystonia, aberrant growth velocity, and skeletal abnormalities. This case demonstrates an unusual pattern of patchy mucosal fibrosis throughout the gastrointestinal tract, alongside duodenal and rectal strictures, with only mild inflammation. While serial endoscopic dilatations and systemic corticosteroids resulted in symptomatic benefit, trial of a Janus kinase‐1 inhibitor (upadacitinib) did not prevent the development of a subsequent rectal stricture. As the pathogenesis of SS remains undefined and treatment modalities are experimental, the observed fibrostenotic complications may point towards new underlying disease mechanisms.

## 1 INTRODUCTION

Satoyoshi syndrome (SS) is a rare, progressive, multisystem disease with proposed autoimmune etiology, featuring alopecia, painful muscle spasms, diarrhea, and skeletal abnormalities.^1^ Corticosteroids, immunoglobulin therapy, or other immunosuppressants have variable response.^2^ We present a novel case of gastrointestinal strictures in SS, and additionally, the first detailed and illustrative report of pathological findings in a living patient with SS. Given the unknown pathogenesis of SS, the fibrostenotic disease behaviour observed in this case may offer valuable insight into potential underlying mechanisms.

## 2 CASE REPORT

A 10‐year‐old female with alopecia universalis, short stature, painful muscle spasms, and chronic diarrhea, presented with recurrent non‐bilious vomiting, retching, bloating, postprandial abdominal pain, and a 5 kg weight loss over 2 years. Examination noted a nondysmorphic, developmentally appropriate child with a distended, tender abdomen, with height 124.4 cm (z‐score −2.88), weight 22.9 kg (z‐score −2.37), and BMI 14.8 kg/m^2^ (z‐score −1.26).

**Figure 1:**
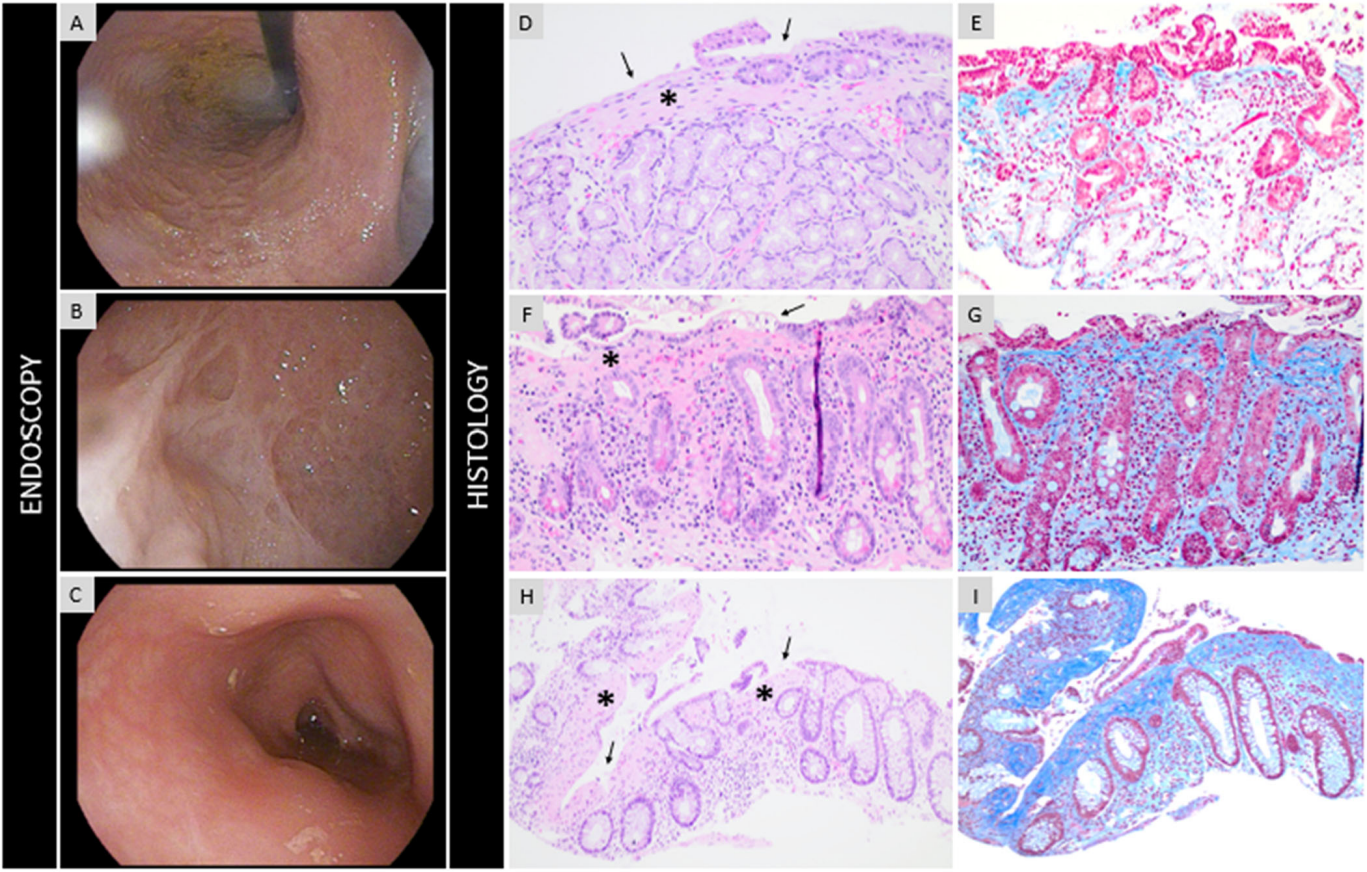
Endoscopy and histology. Stomach (A) and dilated duodenal bulb (B) with pale, irregular mucosa with web‐like appearance. Edematous, non‐friable mucosa in the recto‐sigmoid colon (C). Microscopic images of gastric (D, E), duodenal (F, G), and rectal biopsies (H, I), show patchy fibrosis of the superficial lamina propria (*, H&E; D&F x200, H x100 original magnification) with entrapped scattered inflammatory cells (eosinophils, lymphocytes and plasma cells) and focal degeneration and separation of the surface epithelium (arrows). The band like collagen deposition is highlighted in blue (Masson trichome stain, E & G x200, I x100 original magnification).

**Figure 2:**
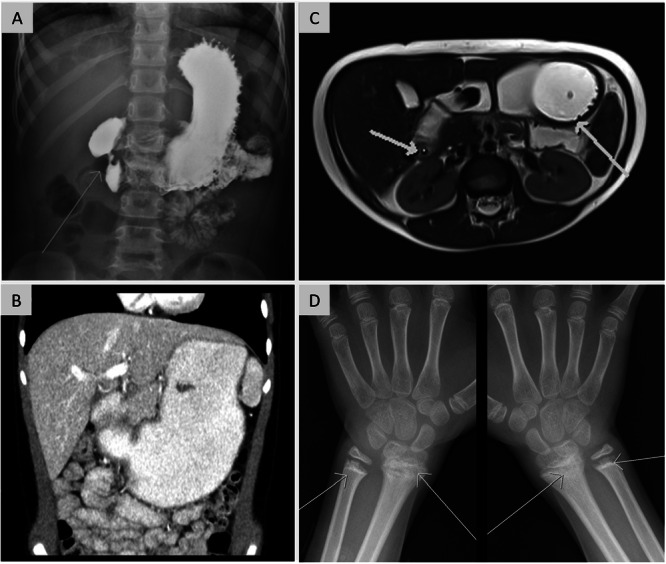
Radiographic investigations. (A) Upper GI barium study showing marked narrowing in the second part of the duodenum (arrow) and mucosal fold thickening in the stomach. (B) Coronal CT image showing a large stomach and wall thickening in the proximal duodenum. (C) Axial HASTE MR image showing wall thickening in the duodenum (short arrow) and gastric fundus (long arrow). (D) Hand x‐ray showing metaphyseal irregularity and sclerosis in bilateral distal radius and ulna.

Despite a strict gluten‐free diet initiated before referral, prompted by villous blunting, crypt hyperplasia, and focally increased intraepithelial lymphocytes in prior duodenal biopsies, celiac serology remained weakly positive (3–4x ULN). Laboratory investigations showed mild hypoalbuminemia, microcytic anemia, a nonspecific auto‐antibody profile, elevated fecal calprotectin, and normal pituitary function (Table 1). A very distended stomach and a severe duodenal stricture with pale, irregular, web‐like mucosa was identified endoscopically and by imaging (Figures 1 and 2). Biopsies showed patchy superficial lamina propria fibrosis with entrapped inflammatory cells and crypt/glandular dilation in the stomach, duodenum, and rectum (Figure 1). Radiographs revealed bilateral widening of physes with metaphyseal irregularities in the distal radius and proximal humerus (Figure 2).

**Table 1:** Investigations in a pediatric case of Satoyoshi syndrome.

Investigation	Result (reference range)
Hemoglobin	109 (110–157 g/L)
Mean corpuscular volume	79 (75–91 fL)
C‐reactive protein	3.4 (0.0–8.0 mg/L)
Albumin	28 (30–45 g/L)
Total protein	57 (62–82 g/L)
Fecal calprotectin	995.9 mg/kg (<50: normal, >250: abnormal)
Anti‐tissue transglutaminase antibody	58 (0.0–14.9 kIU/L)
Anti‐endomysial antibody	Negative
Human leukocyte antigen DQ2/DQ8	DQ2 present; DQ8 not present
Antinuclear antibody	1:40; cytoplasmic speckled pattern
Extractable nuclear antigen antibody profile	
Anti‐Smith	3.6 (<0.9 AI)
Anti‐Smith/ribonucleoprotein	1.0 (<0.9 AI)
Anti‐ribonucleoprotein A	1.5 (<0.9 AI)
Anti‐acetylcholine receptor antibody	2.37 nmol/L (<0.2 nmol/L: absent, 0.2–2 nmol/L: borderline, 2–5 nmol/L: positive low, 5–10 nmol/L: positive medium, >10 nmol/L: positive high)
*Normal liver and renal function, electrolytes, vitamins (A, E, B12, B9), and screening immunodeficiency labs*.	
*Normal pituitary function (thyroid stimulating hormone, bone age, and growth hormone stimulation test)*.	
*Normal pulmonary function test and echocardiogram*.	
*Additional testing was negative for the following antibodies: anti‐double stranded DNA, anti‐glutamic acid decarboxylase 65, anti‐immunoglobulin‐like cell adhesion molecule 5, myopathy/muscle disease profile, and voltage‐gated potassium channel profile*.	
*Whole exome sequencing was non‐diagnostic*.	

During hospital admission, treatment focused on nutritional rehabilitation alongside pharmacologic and endoscopic management. Daily corticosteroids (methylprednisolone for 3 weeks then prednisone tapered over 6 months) and endoscopic pneumatic dilation of the duodenal stricture (6 dilations over 2 months with intralesional triamcinolone injections) resulted in resolution of diarrhea and emesis with improved appetite. Of note, carbamazepine and baclofen failed to control muscle spasms.

Following discharge, despite a 9‐month trial of upadacitinib (7.5 mg daily), gastrointestinal symptoms worsened during prednisone tapering, with development of a new rectal stricture (Supporting Information: Figure 1) and need for additional duodenal dilatation. Biopsies from the stomach and duodenum revealed transient reductions in mucosal inflammation, including eosinophil density and degranulation. However, mucosal fibrosis remained unchanged in the upper gut, while increasing in the distal colon and rectum over time.

Over two‐and‐a‐half years of follow up, weight gain continued (z‐scores −2.5) without linear growth (last z‐score −4.7) on low‐dose prednisone (5 mg daily). Endoscopic appearance of the gastric mucosa improved following resolution of the duodenal stricture, however, leukoplakia and distinctive webbing in the duodenal mucosa persisted (Supporting Information: Figure 2).

## 3 DISCUSSION

The unique constellation of gastrointestinal, musculoskeletal, and dermatologic manifestations, without an alternative gastrointestinal, neurologic, genetic or immune condition, led to the diagnosis of SS.^3^, ^4^

Endoscopic reporting is limited given the rarity of this condition, however upper endoscopic findings of pale, atrophic, irregular gastric mucosa along with duodenal leukoplakia have been described,^3^ consistent with this case. Notably, although a systematic review suggests that stenotic or fibrotic changes are not seen in SS,^3^ the presence of gastrointestinal strictures in this patient suggests they may represent a disease complication. Furthermore, the novel reported endoscopic finding of lacy, web‐like duodenal mucosa in this case appears distinct, and consistent with “mesh‐like” duodenal mucosal changes reported previously on fluoroscopy in SS.^5^ Finally, while our case identified rectosigmoid edema, colonoscopy reporting in SS remains limited, with 5 of 8 cases reporting normal findings.^3^

Very few reports provide detailed descriptions or illustrations of gastrointestinal histopathological findings in SS.^3^ Nonspecific findings, including lymphoplasmacytic and eosinophilic lamina propria infiltrates are most frequently reported in upper gastrointestinal biopsies. Although the fibrotic pattern with crypt dilation and surface epithelial degeneration noted in our case is similar to collagenous colitis, its extension into the muscularis mucosa with band‐like myofibroblast proliferation is atypical and not characteristic of collagenous colitis.

The stromal fibrosis with villous and crypt/glandular distortion in this case were strikingly similar to that reported in an older female with SS who developed colonic adenocarcinoma.^6^ Further histologic findings from three autopsy cases include cystic dilation of glands/crypts throughout the gastrointestinal tract with submucosal herniation, and associated fibrosis without significant associated inflammation, and likely represent more severe/advanced disease.^3^, ^6^, ^7^ Moreover, an adult case of SS with recurrent pancreatitis was deemed to be secondary to duodenal papillary stenosis related to extensive gastrointestinal involvement with associated fibrosis.^7^ The development of multiple gastrointestinal strictures without severe mucosal inflammation alongside the lamina propria fibrosis pattern observed in our case represent a unique phenotype within the SS spectrum.

Several auto‐antibodies have been described in SS, with this patient having low‐titer positivity for antinuclear antibodies, acetylcholine receptor antibodies, and anti‐tissue transglutaminase antibodies, the latter likely nonspecific and not previously reported.^1^, ^3^ Whole exome sequencing was negative for mutations associated with systemic fibrotic disorders. Notably, testing was negative for the homozygous variant in *ZNF808*, a gene of interest in one patient with SS which encodes a zinc‐finger nuclear protein with one Kruppel‐associated box domain, with possible role in immune tolerance.^1^ Underlying genetic mechanisms remain unknown.

SS is usually progressive despite variable reported responses to corticosteroids, intravenous immunoglobulin, and immunomodulators.^2^ In other fibrostenotic gastrointestinal diseases such as Crohn's disease (CD), stricture pathogenesis involves fibroblasts, myofibroblasts, and smooth muscle cells in a complex interplay with immune cells, ultimately leading to fibrosis.^8^ In SS (a condition with substantially less inflammation than seen in CD), the pro‐fibrogenic pathways, potentially involving eosinophil recruitment and pro‐fibrogenic cytokine production, are unknown. Upadacitinib (for concurrent treatment of alopecia areata^9^) was trialed in this case because of the low inflammatory gastrointestinal component, but with the hope of modulating Janus kinase‐mediated downstream signaling events potentially related to fibrosis, reported both clinically and in experimental models.^10^, ^11^ Unfortunately, it improved neither alopecia nor stricture formation despite a transient decrease in eosinophilia histologically. Upadacitinib may not have addressed pathways driving the pro‐fibrotic cascade in this patient, or it may have been given at suboptimal doses. In any case, further work is required to define the pathways leading to fibrosis in patients with this syndrome, which may well differ from those in other intestinal diseases.

## 4 CONCLUSION

This case highlights the ongoing need for continued investigation into the mechanisms of inflammation and disordered fibrogenesis in SS, with the goal of identifying more effective disease‐modifying therapies.

## CONFLICT OF INTEREST STATEMENT

The authors have no conflict of interest.

## ETHICS STATEMENT

Informed patient consent was obtained from the parent of the subject of the case report for publication of the case details.

## Supporting information


**Supplemental Digital Content 1.** Rectal stricture post dilatation identified one year after initial identification of duodenal stricture.

**Supplemental Digital Content 2.** Follow‐up endoscopy at one year following diagnosis of Satoyoshi Syndrome demonstrating duodenal leukoplakia.
