# Supplementary material for: Gastrointestinal strictures in a pediatric patient with Satoyoshi syndrome
Source: JPGN Rep. 2025 Dec 12;7(2):247–51. doi: 10.1002/jpr3.70128 (PMC13150974; doi:10.1002/jpr3.70128)
Supplement: Supplementary file 1 — Supplemental Digital Content 1. Rectal stricture post dilatation identified one year after initial identification of duodenal stricture. [file JPR3-7-247-s002.pdf]

Supplemental Digital Content

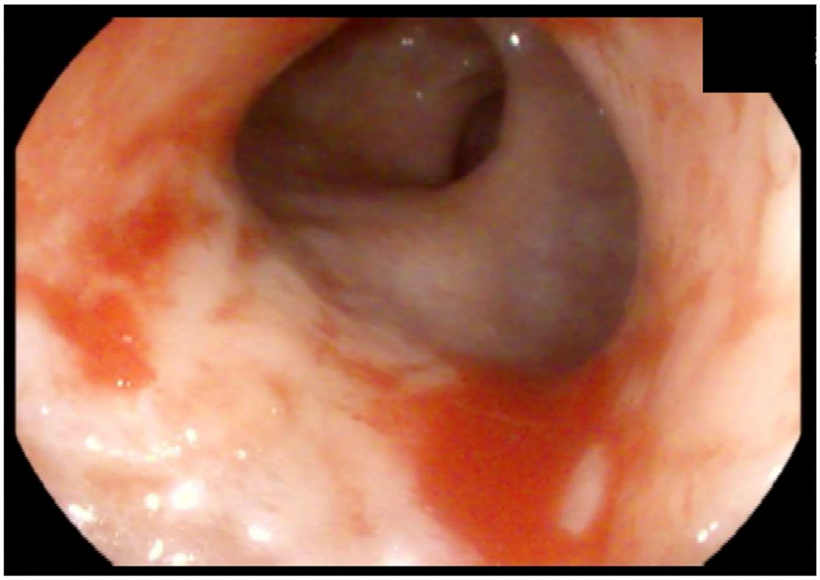

Figure 1, Supplemental Digital Content 1. Rectal stricture post dilatation identified one year after initial identification of duodenal stricture.
