# Supplementary material for: Gastrointestinal strictures in a pediatric patient with Satoyoshi syndrome
Source: JPGN Rep. 2025 Dec 12;7(2):247–51. doi: 10.1002/jpr3.70128 (PMC13150974; doi:10.1002/jpr3.70128)
Supplement: Supplementary file 2 — Supplemental Digital Content 2. Follow‐up endoscopy at one year following diagnosis of Satoyoshi Syndrome demonstrating duodenal leukoplakia. [file JPR3-7-247-s001.pdf]

Supplemental Digital Content

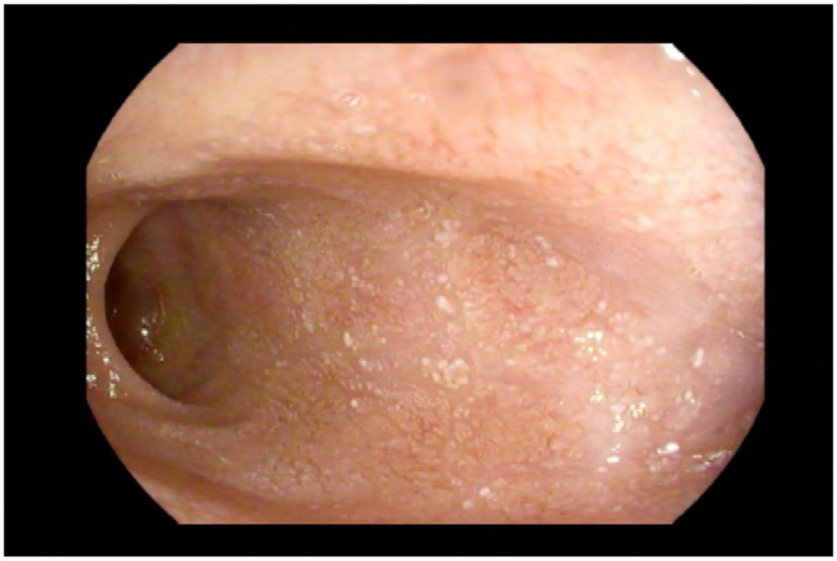

Figure 2, Supplemental Digital Content 2. Follow-up endoscopy at one year following diagnosis of Satoyoshi Syndrome demonstrating duodenal leukoplakia.
